# The Condition Known as "Surgical Kidney"

**Published:** 1909-07-24

**Authors:** 


### Surgery.

### THE CONDITION KNOWN AS "SURGICAL KIDNEY."

### Definition.

The title of "surgical kidney" has long been used to denote a condition in which symptoms referable to disease of the kidney make their appearance secondarily to other surgical conditions, but principally obstructive or paralytic lesions in the lower urinary tract.

The kidney is liable to a number of affections which are the outcome of one or both of two main factors, which are (1) obstruction to the outflow of urine and (2) infection either from without or from the blood stream. The actual condition found will vary in each case according to which of these factors is the more powerful. Sometimes, however, only one of these causes is present. Thus obstruction alone gives rise to hydronephrosis, though it is always possible that infection may occur later and a typical pyonephrosis result; and, again, infection of the kidney without obstruction will most probably cause a condition of acute nephritis.

But where the two causes are present at one and the same time, the result will depend on the relative proportion of infection and obstruction. Thus where the obstructive element outweighs the infective a pyonephrosis is likely to occur, but when these are equivalent pyelonephritis is most often set up, and this is typically the condition to which the name of surgical kidney has been applied.

Pyelonephritis must be regarded then as the result of an ascending inflammation which is secondary to any condition which prevents the natural outflow of urine. It must not be thought that the name implies that the condition is the result of surgical interference; this is not the case.

The common causes of obstruction to the outflow of urine are situated below the neck of the bladder; they are urethral stricture, whether this be traumatic or gonorrhœal in origin, and enlargement of the prostate. Any morbid condition which prevents the bladder from emptying itself, such as disease of or injury to the lumbar spine at the level of the bladder centre will have the same effect. In these cases the renal condition will be bilateral. But pyelonephritis can also occur in a single kidney from obstruction to the passage of urine along one ureter, as, for instance, by the impaction of a calculus in it.

The bladder condition probably acts upon the kidney in more ways than one: (1) the whole genito-urinary tract is constantly irritated, and therefore in a state of persistent hyperæmia, resulting in interstitial nephritis, and (2) as the result of the obstruction the urine is retained under pressure in the pelvis of the kidney which gradually becomes distended; and as the pressure is distributed backwards to the renal substance, the kidney itself is damaged and in time becomes reduced to a collection of cysts.

### Origin of the Infection.

But where does the infective element come from? It has been suggested that this is always the result of the passage of a catheter which is not strictly aseptic. There is no doubt that this occasionally occurs, but that there is ample evidence that it cannot be held responsible for all cases, since pyelonephritis is sometimes met with in cases on which instrumentation has never been performed.

If the outflow from the bladder has been obstructed for any length of time, as, for instance, in a case of enlargement of the prostate, the urine which is withdrawn by catheterisation will be found, if examined microscopically, to contain a number of micro-organisms, any one of which may have the power of ascending the ureters and setting up more or less acute inflammatory changes on the kidney. One kidney is usually affected before the other, but since in the vast majority of cases the obstruction is situated below the neck of the bladder, the condition becomes bilateral sooner or later; but, even then, it will be found post-mortem that one kidney is in a more advanced state of degeneration than the other.

From what has been said, it will be seen that, clinically speaking, the condition is one that should be guarded against by prophylactic measures, since it is hopeless to expect a complete cure when once it has declared itself. In all cases, then, in which there is obstruction to the outflow of urine, two obvious duties confront the surgeon. One is to restore the lumen of the urinary passage with the least possible delay, and the second is to take scrupulous care to keep the interior of the bladder clear by irrigating it with bland antiseptic lotions.

### Symptoms and Signs.

The disease shows itself insidiously. The patient may at first complain of symptoms which have no direct reference to the kidney, such as anorexia or dyspepsia and sleeplessness; and at the same time a slight rise of temperature may be observed. Later he will complain of pain in one or both loins. The urine, which may at first be acid, becomes alkaline later. If examined microscopically it will be found to contain a small amount of pus, albumen, and renal epithelial cells and casts. Examination of the abdomen will reveal a tenderness on palpation in one or both loins, but there will not be any palpable enlargement of the kidney unless the condition has gone on to a pyonephrosis.

The preliminary symptoms should be sufficient to awaken suspicion that the kidney is already damaged, and if the patient is not already in bed he should at once be put there. The obstruction to the outflow of urine should be removed. This is most important, and it is better even to perform a suprapubic cystotomy as a temporary measure than to risk any delay. The bowels should be kept freely open. Drugs should be prescribed which have a diuretic effect as well as a local antiseptic action on the urinary tract. The writer has obtained good results from the use of helmitol, grains xv., three times a day. In addition the bladder should be sedulously irrigated. A good prognosis, however, can hardly ever be given.

